# Non-invasive quantification of human swallowing using a simple motion tracking system

**DOI:** 10.1038/s41598-018-23486-0

**Published:** 2018-03-23

**Authors:** Hiroaki Hashimoto, Masayuki Hirata, Kazutaka Takahashi, Seiji Kameda, Yuri Katsuta, Fumiaki Yoshida, Noriaki Hattori, Takufumi Yanagisawa, Jason Palmer, Satoru Oshino, Toshiki Yoshimine, Haruhiko Kishima

**Affiliations:** 1 Endowed Research Department of Clinical Neuroengineering, Osaka University, Global Center for Medical Engineering and Informatics, Suita, 565-0871 Japan; 2 Department of Neurosurgery, Osaka University Graduate School of Medicine, Suita, 565-0871 Japan; 3 Department of Organismal Biology and Anatomy, University of Chicago, Chicago, 60637 USA; 4 Department of Rehabilitation, Wakakusa Tatsuma Rehabilitation Hospital, Daito, 574-0012 Japan; 5 Department of Anatomy and Neuroscience, Kyushu University Graduate School of Medical Sciences, Fukuoka, 812-8582 Japan; 6 Department of Neurology, Osaka University Graduate School of Medicine, Suita, 565-0871 Japan

## Abstract

The number of patients with dysphagia is rapidly increasing due to the ageing of the population. Therefore, the importance of objectively assessing swallowing function has received increasing attention. Videofluoroscopy and videoendoscopy are the standard clinical examinations for dysphagia, but these techniques are not suitable for daily use because of their invasiveness. Here, we aimed to develop a novel, non-invasive method for measuring swallowing function using a motion tracking system, the Kinect v2 sensor. Five males and five females with normal swallowing function participated in this study. We defined three mouth-related parameters and two larynx-related parameters and recorded data from 2.5 seconds before to 2.5 seconds after swallowing onset. Changes in mouth-related parameters were observed before swallowing and reached peak values at the time of swallowing. In contrast, larynx-related parameters showed little change before swallowing and reached peak values immediately after swallowing. This simple swallow tracking system (SSTS) successfully quantified the swallowing process from the oral phase to the laryngeal phase. This SSTS is non-invasive, wireless, easy to set up, and simultaneously measures the dynamics of swallowing from the mouth to the larynx. We propose the SSTS for use as a novel and non-invasive swallowing assessment tool in the clinic.

## Introduction

The standard clinical examination for swallowing includes videofluoroscopy (VF) and videoendoscopy (VE)^1^. These methods are considered the gold standards for evaluating swallowing function because they directly provide visual information about swallowing movement and the corresponding clinical metrics. However, they have the disadvantages of being bulky and invasive^2^. Furthermore, VF exposes patients to radiation. These methods are not suitable for routine use, do not easily quantify swallowing-related movement, and utilize controversial clinical metrics.

Several types of non-invasive methods for evaluating swallowing function, including sound sensors^1–4^, respiratory flow^4^, electromyograms (EMG)^5^, accelerometers^1,3^, electroglottographs (EGG)^1,6^, ultrasound^7,8^, and mechanomyography (MMG)^9^, have been reported. These methods provide on-sensor measurements that require the placement of sensors on participants’ skin, and for some patients who suffer from dysphagia, the placement of these sensors is cumbersome if not unfeasible. A method designed to measure swallowing using magnetic resonance imaging (MRI) was reported^10–12^, but this method is bulky. In the present study, we developed a novel, non-invasive, off-sensor, quantitative tool using a popular wireless motion tracking system, the Kinect v2 sensor (Microsoft, Redmond, Washington, USA), to quantify swallowing-related motion in healthy participants. We designated this tool as the simple swallow tracking system (SSTS). The Kinect v2 sensor enables users to easily obtain quantitative motion data. We non-invasively monitored patients’ swallowing with both EGGs and throat microphones to detect swallowing onset for confirmation. Simultaneously, the SSTS was used to register the real-time and non-invasive three-dimensional (3D) movements of the mouth and larynx related to swallowing. Here, we present evaluations of mouth and larynx movements related to swallowing using the SSTS. The advantages of this method are its non-invasiveness, its wireless nature, and the simplicity of setup, which may enable its use at the bedside. Our eventual goal is the clinical application of our SSTS for the quantification of swallowing.

## Results

The 3D features of swallowing movements were recorded with our SSTS. We defined five swallowing-related parameters, including three for mouth movements and two for laryngeal movements. We defined mouth width (MW), mouth openness (MO), and lip protrusion (LP) as the mouth-related parameters and vertical motion (VM) and horizontal motion (HM) as the larynx-related parameters.

Table 1 shows the average values and standard deviations (SDs) for each parameter among all participants during measurements. These results were calculated from the total recorded data, which were not time-locked to swallowing. Before performing these calculations, MW, VM, and HM outliers were excluded (see the data analysis section) (Supplementary Fig. S1). Significant differences in age (*p* = 0.153, two-tailed t-test) or the number of swallows (*p* = 0.938, two-tailed t-test) were not observed between males and females. However, significant differences in MW (*p* = 0), MO (*p* = 0), LP (*p* = 0), VM (*p* = 4.99 × 10^−107^), and HM (*p* = 0) were observed between the male and female groups, according to the Wilcoxon rank sum test (Supplementary Fig. S2). Among the five males, significant differences in MW (*p* = 0), MO (*p* = 0), LP (*p* = 0), VM (*p* = 0), and HM (*p* = 0) were observed, according to the Kruskal-Wallis test (Supplementary Fig. S3). Among the five females, significant differences in MW (*p* = 0), MO (*p* = 0), LP (*p* = 0), VM (*p* = 0), and HM (*p* = 0) were also observed, according to the Kruskal-Wallis test (Supplementary Fig. S4). We identified considerable inter-gender and inter-individual variances and consequently did not directly compare raw data within parameters or between parameters. Therefore, the data were time-locked to 2.5 seconds (s) before and after swallowing and z-normalized using the average values and SDs presented in Table 1 for each parameter in each participant (Supplementary Fig. S1d). This normalization strategy enabled us to statistically compare the swallowing-related parameters between groups or within groups.

**Table 1:** Values of each parameter for each participant. Five males and five females participated in this study. The average values and SDs were calculated using the total data for each parameter.

Participant	Sex	Age	Swallowing number	Mouth width (MW)	Mouth openness factor (MO)	Lip protrusion factor (LP)	Vertical motion (VM)	Horizontal motion (HM)
1	M	30	24	0.38 ± 0.03	0.08 ± 0.05	0.39 ± 0.15	−2.43 ± 1.02	−59.16 ± 20.99
2	M	39	25	0.41 ± 0.02	0.15 ± 0.07	0.19 ± 0.19	−7.64 ± 1.59	−59.58 ± 14.58
3	M	32	41	0.42 ± 0.03	0.15 ± 0.24	0.12 ± 0.12	−5.74 ± 1.35	−61.91 ± 21.33
4	M	32	42	0.44 ± 0.05	0.20 ± 0.21	0.38 ± 0.13	−5.78 ± 0.96	−41.18 ± 11.73
5	M	35	32	0.41 ± 0.03	0.29 ± 0.07	0.19 ± 0.20	−4.33 ± 0.74	−23.94 ± 14.01
Total males		33.6 ± 3.51	32.8 ± 8.53	0.41 ± 0.03	0.16 ± 0.17	0.21 ± 0.19	−5.92 ± 2.01	−55.08 ± 20.90
11	F	36	41	0.36 ± 0.04	0.08 ± 0.09	0.20 ± 0.13	−15.07 ± 0.83	−49.12 ± 12.90
12	F	18	41	0.37 ± 0.03	0.38 ± 0.23	0.51 ± 0.11	−0.50 ± 0.61	−27.69 ± 17.36
13	F	29	30	0.39 ± 0.03	0.08 ± 0.13	0.57 ± 0.22	−4.84 ± 1.27	−24.83 ± 25.10
14	F	32	26	0.37 ± 0.02	0.10 ± 0.14	0.74 ± 0.16	−4.17 ± 1.31	−36.29 ± 27.36
15	F	26	28	0.39 ± 0.03	0.09 ± 0.12	0.55 ± 0.18	−7.21 ± 1.04	−12.63 ± 15.72
Total females		28.2 ± 6.80	33.2 ± 7.26	0.38 ± 0.03	0.13 ± 0.18	0.51 ± 0.23	−6.35 ± 5.40	−30.19 ± 23.42
All		30.9 ± 5.84	33.0 ± 7.47	0.40 ± 0.04	0.15 ± 0.18	0.32 ± 0.25	−6.06 ± 3.48	−47.07 ± 24.66

**Figure 1:**
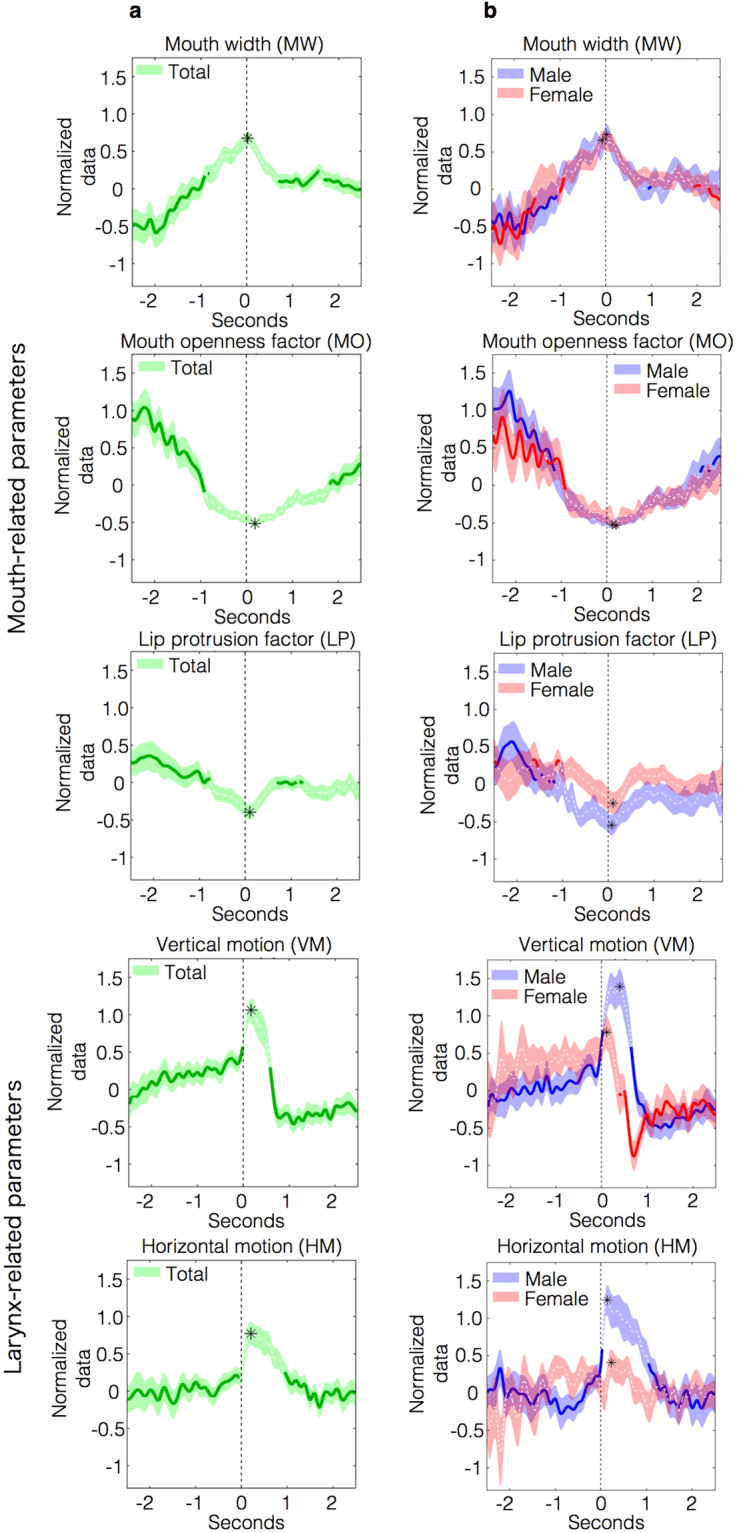
Graphs of mouth-related parameters (MW, MO, and LP) and larynx-related parameters (VM and HM) from 2.5 s before to 2.5 s after swallowing onset. MW, MO, and LP were the mouth-related parameters. VM and HM were the larynx-related parameters. Error bars indicate 95% CIs. Zero seconds corresponds to the onset of swallowing. The positive peak values for MW, VM, and HM and the negative peak values for MO and LP are indicated with asterisks. Peak values were compared with other data points using one-way ANOVA and a multiple comparisons test. Significantly different data points (corrected *p* < 0.01) are shown as solid lines. (**a**) Results from the total group are shown. The mouth movement parameters started to change before swallowing and reached positive peak values or negative peak values at the time of swallowing. In contrast, the larynx-related parameters showed little change before swallowing but reached positive peak values immediately after swallowing. Peak values were significantly different from the data points collected before or after swallowing. (**b**) Results from both the male and female groups are displayed. For MW, MO, and LP, the graphs displayed similar trends for males and females; however, for VM and HM, differences were observed between males and females from 0 to 1.0 s. The changes in VM after swallowing were more notable in males than in females. In females, the peak values for HM were not significantly different from the other data points.

Using normalized data, we obtained averaged waveforms (Fig. 1 and Supplementary Fig. S1d) and their 95% confidence intervals (CIs) 2.5 s before and after swallowing onset to delineate the temporal profiles of the normalized swallowing-related parameters. Figure 1 presents the results from the three groups, comprising the total, male, and female participants.

### Mouth-related parameters

Changes in mouth-related parameters began to be observed before swallowing and exhibited positive or negative peaks at the time of swallowing. Mouth closing, widening of the mouth, and flattening of the lip occurred simultaneously at the time of swallowing. At −2.0 s, a positive value for MO indicated mouth opening. At approximately −1.0 s, zero values for MW, MO, and LP indicated that the mouth had returned to its initial position. Then, the MW increased and peaked at the time of swallowing. In contrast, MO and LP decreased and exhibited negative peaks at the time of swallowing. MW, MO, and LP showed similar temporal profiles among total, male, and female groups (Fig. 1).

### Larynx-related parameters

Larynx-related parameters did not show clear changes before swallowing, but showed immediate changes after swallowing (Fig. 1a). Videos from the SSTS showed that these changes were caused by a positional shift of the thyroid cartilage (Supplementary Video S1). Before swallowing, values for VM and HM were approximately zero, but these parameters exhibited a sharp increase at swallowing onset.

VM and HM exhibited differences between males and females; no overlap in 95% CIs was observed (from approximately 0.1 to 1.0 s for VM and from 0 to 1.0 s for HM) (Fig. 1b). In males, both VM and HM showed sudden, sharp increases immediately after swallowing onset (0 s) that peaked within 0.5 s after swallowing onset. Then, both VM and HM decreased to their initial values. In females, VM also increased after swallowing onset, but the increase was much smaller than that in males. Soon after VM peaked, it immediately decreased and exhibited a negative peak with a clear trough. VM then returned to its initial position, as it did in males. In females, HM did not exhibit apparent changes either before or after swallowing.

### Statistical analyses of peak values related to swallowing

We observed positive peak values for MW, VM, and HM and negative peak values for MO and LP in both male and female groups. The data points of the peaks are indicated as asterisks (Fig. 1a,b). For the statistical analysis, the data points of the positive and negative peaks were compared with the remaining data points (one-way ANOVA and a multiple comparison test, corrected *p*-value < 0.01). The data points that displayed significant differences are shown as solid lines (Fig. 1a,b). In the total group, significant differences were observed in the peak data points for MW (corrected *p* = 2.23 × 10^−37^ to 0.00868), MO (corrected *p* = 1.72 × 10^−58^ to 0.00424), LP (corrected *p* = 6.56 × 10^−17^ to 0.00831), VM (corrected *p* = 8.53 × 10^−50^ to 6.24 × 10^−6^), and HM (corrected *p* = 1.41 × 10^−18^ to 0.00543) before or after swallowing. In the male group, significant differences were observed in the peak data points for MW (corrected *p* = 1.03 × 10^−16^ to 0.00953), MO (corrected *p* = 1.09 × 10^−35^ to 0.00986), LP (corrected *p* = 1.41 × 10^−15^ to 0.00646), VM (corrected *p* = 2.05 × 10^−46^ to 8.77 × 10^−4^), and HM (corrected *p* = 9.59 × 10^−22^ to 0.00724). In the female group, the peak data points for MW (corrected *p* = 7.42 × 10^−26^ to 0.00795), MO (corrected *p* = 1.26 × 10^−19^ to 0.00510), LP (corrected *p* = 6.12 × 10^−5^ to 0.00766), and VM (corrected *p* = 1.04 × 10^−27^ to 0.00658) also showed significant differences. However, the peak HM in females was not significantly different from the other data points obtained before and after swallowing. The peak values for the remaining parameters during swallowing were significantly different from the other data points collected before and after swallowing.

## Discussion

In the present study, we developed the SSTS using the Kinect v2, which is non-invasive, wireless and able to quantitatively and simultaneously record dynamic movements of the mouth and larynx. The oral phase of swallowing recruits the jaw-closing muscles of the mandible to stabilize the mandible^13^. Additionally, the orbicularis oris and buccinator muscles firmly close the mouth to prevent food from escaping, flatten the cheeks and hold the food in contact with the teeth^13,14^. The mouth-related parameters observed in this study clearly capture the three-dimensional features of sequential mouth movements during swallowing. First, the mouth opened for water intake. Then, the mouth closed and stretched laterally, and the lip protrusion flattened for swallowing. After swallowing, the mouth returned to its initial position.

**Figure 2:**
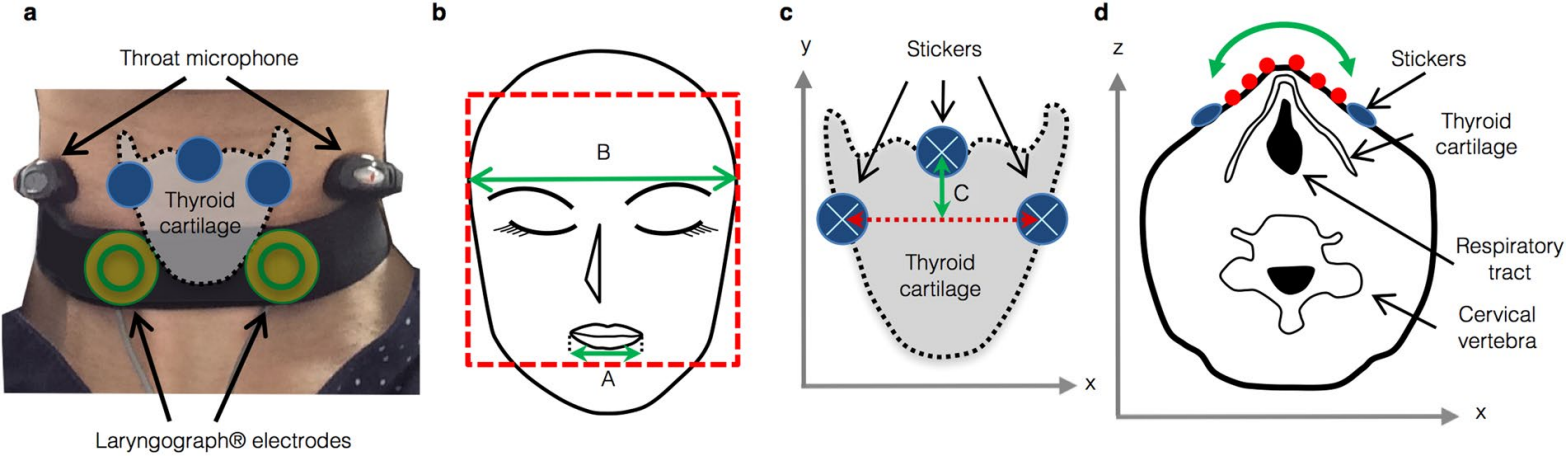
Schematic of non-invasive swallowing measurements and parameters recorded by the SSTS. (**a**) We monitored swallowing with electroglottography (EGG, laryngograph) and a throat microphone. A pair of laryngograph electrodes was placed below the thyroid cartilage. The microphone was placed around a participant’s neck. Three blue stickers were attached to the thyroid cartilage for recognition by the Kinect v2 sensor to monitor laryngeal movements. (**b**) MW is the parameter involved in mouth movement and is defined as the ratio of the width between the corner edges of the mouth (A) to the width of the face (B). MW = A/B. (**c**) VM is the difference in y coordinates between the median stickers and the averaged outside stickers in the xy plane. VM = C. VM indirectly represented vertical laryngeal motion. (**d**) Schematic of an axial laryngeal slice. The surface between the outside stickers was approximated to a convex quadratic function in the xz plane (green curved line), and the associated quadratic coefficient was HM. HM represented the degree to which a participant’s larynx projects forward. HM indirectly indicated horizontal laryngeal motion. Red circles indicate pixels available for the calculation of HM in the xz plane.

The larynx-related parameters did not reveal clear changes before swallowing, but it was suddenly elevated immediately after swallowing onset and then returned to its initial position. The thyroid cartilage elevates during swallowing, thus preventing material from entering the tracheal airway^1^ and clearing a bolus^15^. The larynx-related parameters in the present study, particularly VM, clearly captured this elevation. The sticker placed in the middle of the laryngeal prominence is effectively displaced relative to stickers placed outside this structure due to thyroid cartilage movement. The difference in VM between genders may be caused by anatomical differences in the thyroid cartilage. The thyroid cartilage is larger and the breadth of the thyroid lamina is significantly wider in males than in females^16,17^. HM changes were clearly observed in the male group; however, in the female group, HM showed no clear changes during swallowing. HM also reflects the degree of protrusion of laryngeal projections (Fig. 2d). Therefore, we inferred that this difference between genders also resulted from the anatomical differences in the thyroid cartilage. The thyroid cartilage protrudes to a lesser extent in adult females than in adult males^17^. Based on these differences, the present tracking system may successfully delineate the sex differences in laryngeal movements during swallowing. However, based on VF research, hyoid excursion during swallowing depends on a person’s size rather than sex differences^18^. Moreover, our sample size may be insufficient to analyse significant differences between genders or individuals. Further studies are needed to determine whether the SSTS can identify sexual or individual differences.

In the present study, we successfully quantified swallowing-related movements with the SSTS in healthy participants with normal swallowing function. The advantages of this system are its non-invasiveness, its wireless nature, its unconstrained measurements, and the simplicity of setup. Moreover, this system simultaneously measures both oral and laryngeal movements. According to our results, the initial mouth movement preceded the initial laryngeal movement during swallowing. Our method displays exceptional advantages over VF and other non-invasive swallowing modalities, because VF is invasive and other non-invasive swallowing modalities do not simultaneously measure the dynamics of swallowing from the mouth to the larynx. Our ultimate goal is the clinical application of this system for the quantification of dysphagia. In patients with dysphagia, the dynamics of swallowing from the mouth to the larynx are disturbed to varying extents, depending on the disease^19^, and this system may be able to detect disturbances between the mouth and larynx and quantify the degree of dysphagia. We believe that this SSTS will be useful for quantitatively evaluating the therapeutic effects of dysphagia rehabilitation. For example, this system may be useful for instruction in and the evaluation of the Mendelsohn manoeuvre. The Mendelsohn manoeuvre is intended to be effective for patients with dysphagia, but it is difficult to teach^20^. The SSTS may facilitate the implementation of this manoeuvre because it quantifies swallowing-related kinematics, particularly larynx-related motion. We postulate that this SSTS will provide valuable insights into swallowing mechanisms and complement existing methods for studying deglutition.

However, this study has some limitations. First, we need to analyse differences between genders in a larger sample size because the number of participants in the present study was small. Second, the effects of head, neck, and body motion were not considered when larynx-related parameters were calculated. Considerable body and/or head motion may occur during swallowing^21^. VM may be influenced by body motion, which occurs in the yz plane, because VM is exclusively calculated from coordinates in the xy plane (Supplementary Fig. S5). However, corrections for body motion will be feasible by adding a new fixed sticker on the neck. If we calculate the new x’y’z’ coordinates, which are defined by a fixed sticker and bilateral stickers, changes in the position of the median sticker using the new coordinates will be independent of body motion. Finally, we observed indirect laryngeal movements, because the larynx-related parameters were based on measured movement of the skin instead of directly measuring the movement of the larynx. The larynx is not rigidly attached to the skin and can glide under the skin.

VF is a standard examination for dysphagia because it reveals the nature of and quantifies dysphagia^18,22,23^. However, we have not known whether the SSTS can distinguish the features of dysphagia from normal swallowing. Moreover, this system cannot easily quantify aspiration, particularly silent aspiration, which is clinically crucial and the most severe situation. We must concurrently measure dysphagia using our system and VF to verify this hypothesis.

## Methods

### Non-invasive monitoring of swallowing

1. Laryngograph. The laryngograph (Laryngograph Ltd., London, UK) is an electroglottography (EGG) device mainly used in voice clinics. Laryngograph sensors readily detect impedance changes in the neck caused by vibration of the vocal folds^1^. During swallowing, the vocal cords always close in healthy individuals to prevent aspiration^1,24^. Closure of the vocal cords increases an equivalent cross-sectional area of the route of the electric current and reduces the impedance of the measured region. According to Kusuhara *et al*., the change in neck impedance corresponds to swallowing such that the impedance waveform was easy to read during swallowing activities^6^. As reported in the study by Firmin *et al*., the laryngograph provides a reliable signal relative to swallowing^1^. In the present study, a pair of laryngograph electrodes was placed below the thyroid cartilage at a centre-to-centre distance of 25 mm and was held in place by an elastic band (Fig. 2a). The sampling rate was set to 24,000 Hz. Representative waveforms, which were obtained by additional averaging of signals that were time-locked to the onset of swallowing, from Participant 1 are shown in Fig. 3a. The swallowing onset time was determined visually at the time when the impedance waveform began to increase from baseline.

**Figure 3:**
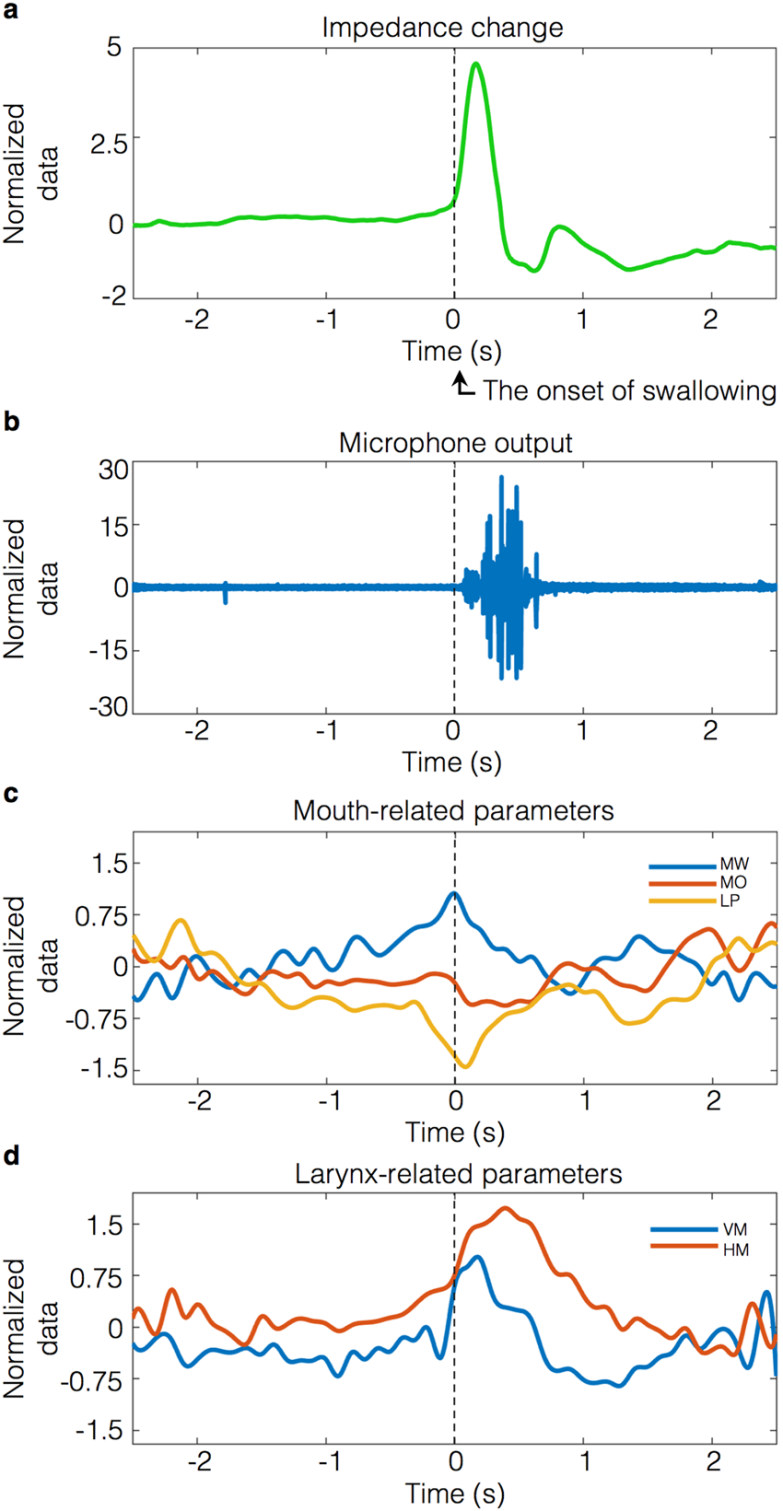
Representative waveforms of Participant 1. These waveforms were traced from a 30-year-old male participant (Participant 1) and were calculated by the additional averaging of signals that were time-locked to the onset of swallowing. (**a**) Signals recorded by the laryngograph (EGG) changed upon swallowing. The onset of swallowing was detected at the initial rise of the waveform. (**b**) The waveforms were recorded by a throat microphone. The sounds of swallowing were caused by a food bolus passing through the pharynx. (**c**) The mouth-related parameters MW, MO, and LP changed during swallowing. These parameters began to change before swallowing and exhibited positive or negative peaks at the onset of swallowing. (**d**) The larynx-related parameters VM (vertical motion) and HM (horizontal motion) did not exhibit appreciable changes before swallowing, but these parameters suddenly exhibited a positive peak immediately after swallowing.
2. Throat microphone. Acoustical methods for the detection of swallowing have been reported. The sounds of swallowing are caused by a bolus passing through the pharynx^6^ and have been recorded by a microphone^2,6^. We connected a throat microphone (Inkou mike; SH-12iK, NANZU, Sizuoka, Japan) to the laryngograph to measure swallowing sounds. When participants swallowed, impedance changes were first measured by the laryngograph. Immediately thereafter, swallowing sounds were recorded (Fig. 3a,b). Sound was apparently not contaminated by myoactivity. We detected the swallowing time by monitoring the onset of impedance changes, because the impedance changes are more sensitive to laryngeal motion than sound. We used swallowing sounds to confirm that the impedance changes indicated true swallowing movement and were not caused by noises, artefacts, or other sources. Additionally, we confirmed that the detected swallowing time corresponded to true swallowing using video captured by the Kinect RGB camera (see below). The shape of the microphone was arched and was placed around the participant’s neck (Fig. 2a). The sampling rate was set to 24,000 Hz. A representative waveform, which was obtained by additional averaging of signals that were time-locked to the onset of swallowing, from Participant 1 is shown in Fig. 3b.
3. Kinect RGB camera. During swallowing, the larynx moves forward and upward for airway protection^1,6,24^ and bolus clearance^15^. We captured the motion of the mouth and larynx with the Kinect v2 RGB camera at a rate of 30 frames per second (fps).
4. An electric stimulator (NS-101; Unique Medical, Tokyo, Japan) supplied synchronizing digital signals at 1 Hz to the laryngograph and caused the LED lights to flash. This flashing was captured by the Kinect RGB camera. We matched the digital signals to the captured lights, enabling us to synchronize all captured data. The multi-modal data were integrated to enable swallowing detection.

### 3D motion features during swallowing

The Kinect v2 also possesses infrared depth sensors that recognize body parts in real time^25^ and simultaneously provide the three-dimensional x, y, and z spatial coordinates (Fig. 2c,d, and Supplementary Fig. S6) of the points of interest. The high-definition face tracking (HDFT) component of the Kinect v2 software development kit (SDK) allows real-time, non-invasive measurements without calibration or training for tracking. In this paper, 3D motion features related to the mouth and larynx during swallowing were captured by Kinect v2 sensors, and we evaluated the features specific to swallowing. We established three mouth-related parameters and two larynx-related parameters. The data recorded by the Kinect v2 were obtained at 30 fps, and the parameters were calculated from only one frame of data.

### Mouth-related movement

MW estimation. The ratio of the width between the corner edges of the mouth to the width of the face was defined as the MW parameter (Fig. 2b). The Kinect v2 recognizes the face as a square element, and we used the width of one side of the square as the width of the face. The Kinect v2 provides the coordinates of the corner edges of the mouth.

Kinect animation unit estimation. The Kinect v2 sensor provides animation units (AUs) that use captured facial movements for HDFT^26^. AUs consist of 17 patterns, and we chose two AUs that may be involved in swallowing. One AU is FaceShapeAnimations_JawOpen, and the other AU is FaceShapeAnimations_LipPucker. We described JawOpen as the MO and LipPucker as the LP. AUs are expressed as a numeric weight ranging between 0 and 1^26^.

## Larynx-related movement

Three stickers were attached to a participant’s larynx and were recognized by the Kinect RGB camera to quantify laryngeal movement during swallowing. One sticker was attached to the laryngeal prominence of the thyroid cartilage along the median line, and the other two stickers were arranged on each side of this sticker. The distance between the median sticker and each lateral sticker was approximately 10 mm (Fig. 2a). Round blue stickers with a diameter of approximately 14 mm were used. We developed a custom image processing programme to detect the centre position of stickers attached to the neck in x, y, and z spatial coordinates.

Quantification of vertical laryngeal motion. The difference between the y coordinate of the median sticker (Y) and the average y coordinate of the outside stickers (AY) was defined in the xy plane as a parameter that indicates the VM of the larynx during swallowing (Fig. 2c) using the following equation (1):

1 $$VM(t)=AY(t)-Y(t).$$

Quantification of horizontal laryngeal motion. The Kinect v2 recorded x and z coordinates on the line segment between the outside stickers. If the number of pixels on the segment was n, n data points were available, $({{\rm{x}}}_{1},{{\rm{z}}}_{1}),\,...,({{\rm{x}}}_{{\rm{n}}},{{\rm{z}}}_{{\rm{n}}})$. The curved surface of the larynx was approximated with a convex quadratic function on the xz plane (Fig. 2d). The quadratic coefficient of the quadratic function was defined as a parameter indicating the HM of the larynx during swallowing. The quadratic coefficient was calculated using the following equation with the least squares method (2):

2 $$HM(t)=\frac{n\Sigma {x}^{2}\Sigma {x}^{2}z-\,\Sigma x\Sigma x\Sigma {x}^{2}z+\,\Sigma x\Sigma {x}^{2}\Sigma xz-n\Sigma {x}^{3}\Sigma xz+\,\Sigma x\Sigma {x}^{3}\Sigma z-\,\Sigma {x}^{2}\Sigma {x}^{2}\Sigma z}{2\Sigma x\Sigma {x}^{2}\Sigma {x}^{3}+\,n\Sigma {x}^{2}\Sigma {x}^{4}-\,\Sigma x\Sigma x\Sigma {x}^{4}-n\Sigma {x}^{3}\Sigma {x}^{3}-\,\Sigma {x}^{2}\Sigma {x}^{2}\Sigma {x}^{2}}$$

in which *Σx* is used to abbreviate ${\sum }_{i=1}^{n}x{(t)}_{i}$.

The Kinect v2 captured videos at a rate of 30 fps, and feature extraction was performed approximately every 33 milliseconds. Motion features were analysed using MATLAB (MathWorks, Natick, MA, USA).

### Participants and tasks

Ten healthy adult volunteers (5 males and 5 females) with normal swallowing function participated in the study. Participants were not obese. In accordance with the Declaration of Helsinki, we explained the purpose and possible consequences of this study to all participants and obtained informed consent prior to participation. The ethics committee of Osaka University Hospital approved the protocol used in this study.

The participants were seated facing the Kinect v2, which was placed on a tripod at a distance of approximately one metre, and were asked to fix their heads along the median line because missed frames predominantly occurred when the head was yawed or pitched^27^. The Kinect v2 was angled to frame the upper bodies of the participants. The examiner injected 2 ml of water into their mouths using a syringe, and the participants swallowed the water bolus at their own pace without external prompting. At that time, we also asked participants to only swallow without other motions. During measurements, the examiner was careful not to raise his hand over a participant’s mouth and larynx to avoid tracking failures^27^.

### Data analysis

MO and LP were data provided directly by the Kinect v2, but MW, VM, and HM were secondary data calculated from the original Kinect data. If the Kinect v2 failed to recognize the face as a square, the MW was inaccurate. Similarly, a failure to recognize the stickers would lead to inaccurate VM and HM values. Therefore, we evaluated the distribution of raw MW, VM, and HM values from −2.5 s before to 2.5 s after swallowing onset and visually determined the upper and lower limits to exclude outliers (Supplementary Fig. S1 and Supplementary Table S1). For MW, VM, and HM, we created total modified data after excluding outliers, and the quantity of excluded data is shown in Supplementary Table S1. Next, we calculated the average values and SDs for each parameter from total raw data for MO and LP and the total modified data for MW, VM, and HM for each participant. Then, all parameters that had been time-locked to the onset of swallowing were z-normalized using each average value and SD for comparison within parameters or groups (Supplementary Fig. S1d).

The participants were divided into three groups containing males, females, or all participants. We analysed the normalized Kinect data that had been time-locked to the onset of swallowing from 2.5 s before swallowing to 2.5 s after swallowing. The Kinect data were obtained approximately every 33 ms (30 fps) for a total of 151 data points per parameter per participant. For each parameter, we calculated the average values and 95% CIs for each of these 151 data points in each group. We calculated a weighted mean value for the swallowing number because the number of swallows differed between participants. The original data plots showed the fluctuations along the time axis (Supplementary Fig. S1d), and we smoothed the graph.

### Statistics

A two-tailed t-test was used to compare age and swallowing number between genders. The data collected during measurements were not normally distributed (Supplementary Figs S2–S4). The Wilcoxon rank sum test was performed to compare the total data measured for each parameter between genders; raw data were used for MO and LP and modified data were used for MW, VM, and HM. The Kruskal-Wallis test was performed for comparisons of total data measured for each parameter between individuals; raw data were used for MO and LP and modified data were used for MW, VM, and HM.

We identified the positive or negative peak values for averaged waveforms calculated from normalized data time-locked to 2.5 s before and after swallowing onset for each parameter in each group. We used one-way ANOVA and a multiple comparisons test to identify significant differences between the peak value and other data points. In our tests, we applied a conservative Bonferroni correction to correct for multiple comparisons and used a threshold of corrected *p* < 0.01.

We compared differences between genders by assessing overlapping or non-overlapping CIs. Non-overlapping CIs were considered an indicator of statistical significance. Overlapping CIs were interpreted to indicate a lack of statistical significance^28^. However, this conservative interpretation might overlook statistically significant differences. The method of examining overlap is more conservative than the standard method when the null hypothesis is true, and it mistakenly fails to reject the null hypothesis more frequently than the standard method when the null hypothesis is false^29^.

### Data availability

All data generated or analysed in this study are available from the corresponding author upon reasonable request after additional ethical approval regarding data provision to individual institutions.

## Electronic supplementary material

Supplemetary Video Movie

Supplemetary Information
