# Supplementary material for: Non-invasive quantification of human swallowing using a simple motion tracking system
Source: Sci Rep. 2018 Mar 23;8:5095. doi: 10.1038/s41598-018-23486-0 (PMC5865167; doi:10.1038/s41598-018-23486-0)
Supplement: Supplementary file 2 — Supplemetary Information [file 41598_2018_23486_MOESM2_ESM.pdf]

Supplementary Information for

Non-invasive quantification of human swallowing using a simple motion tracking system

Hiroaki Hashimoto[1,2], Masayuki Hirata[1,2*], Kazutaka Takahashi[3], Seiji Kameda[1], Yuri Katsuta[4], Fumiaki Yoshida[5], Noriaki Hattori[1,6], Takufumi Yanagisawa[1,2], Jason Palmer[1], Satoru Oshino[2], Toshiki Yoshimine[1], Haruhiko Kishima[2]

1) Osaka University, Endowed Research Department of Clinical Neuroengineering, Global Center for Medical Engineering and Informatics, Suita, 565-0871, Japan.
2) Osaka University Graduate School of Medicine, Department of Neurosurgery, Suita, 565-0871, Japan.
3) University of Chicago, Department of Organismal Biology and Anatomy, Chicago, 60637, USA.
4) Wakakusa Tatsuma Rehabilitation Hospital, Department of Rehabilitation, Daito, 574-0012, Japan.
5) Kyushu University Graduate School of Medical Sciences, Department of Anatomy and Neuroscience, Fukuoka, 812-8582, Japan.
6) Osaka University Graduate School of Medicine, Department of Neurology, Suita, 565-0871, Japan.

Contents:

Supplementary Figures 1-6
Supplementary Table 1
Supplementary Video 1 (separate file)

Supplementary figures

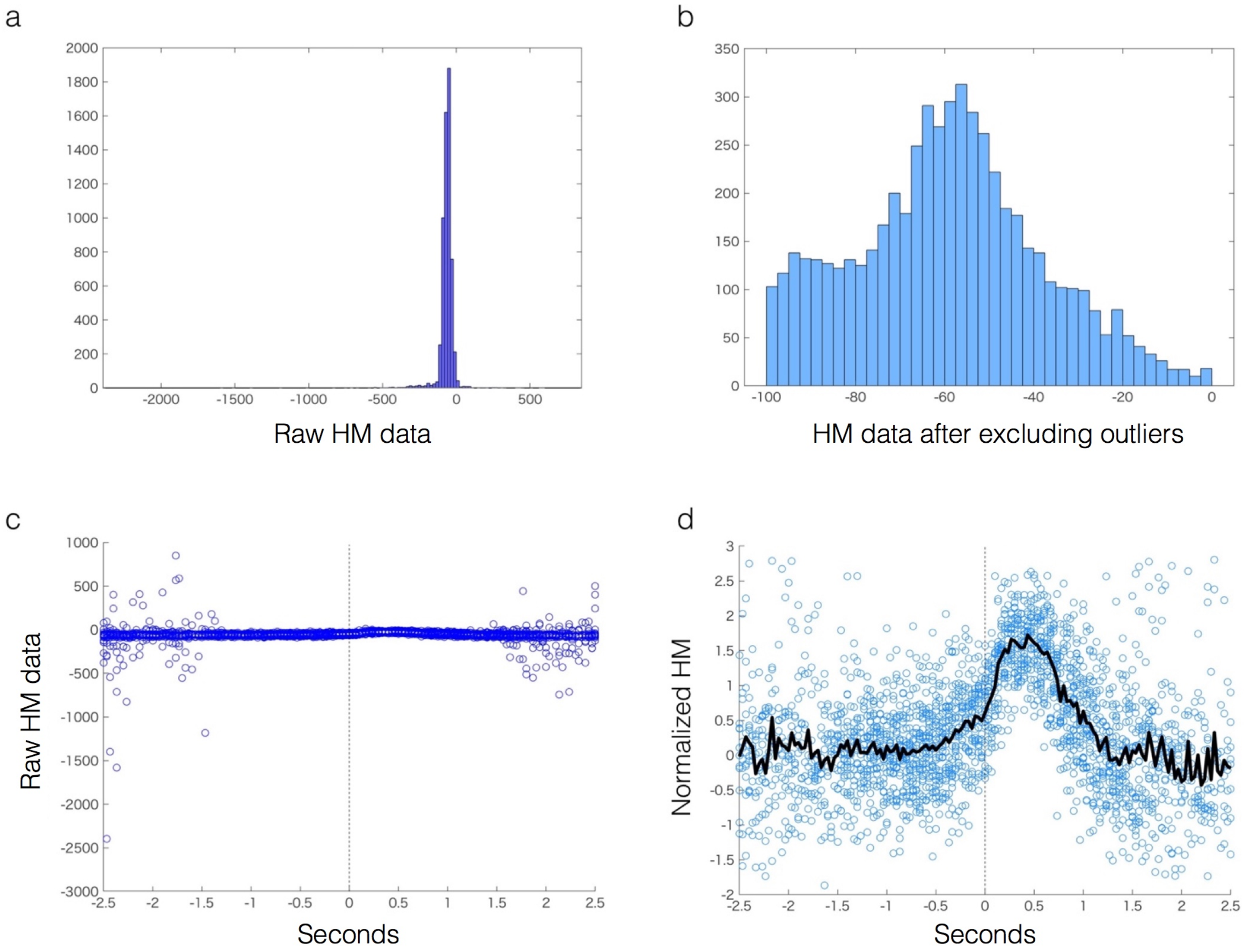

Supplementary Figure S1. Distribution of raw and normalized HM data for Participant 1. (a) The distribution of total raw HM data collected during measurements. (b) The distribution of total HM data after the exclusion of outliers. The upper and lower limits for excluding outliers are shown in Supplementary Table 1. For MW, VM, and HM, the average values and standard deviations shown in Table 1 were calculated based on this modified group. (c) The distribution of raw data time-locked to 2.5 seconds before and after swallowing onset. Zero seconds corresponds to the swallowing onset time. We visually determined the upper and lower limits for the exclusion of outliers using the data presented in this figure. (d) The distribution of normalized data time-locked to swallowing onset. Data were z-scored using the average values and standard deviations shown in Table 1. The black solid line was obtained from the average values of normalized data for each data point. One hundred fifty-one data points were collected in the period 2.5 seconds before and after swallowing. The solid waveform was obtained from one participant, but waveforms in Figure 1 were obtained from five or ten participants per group.

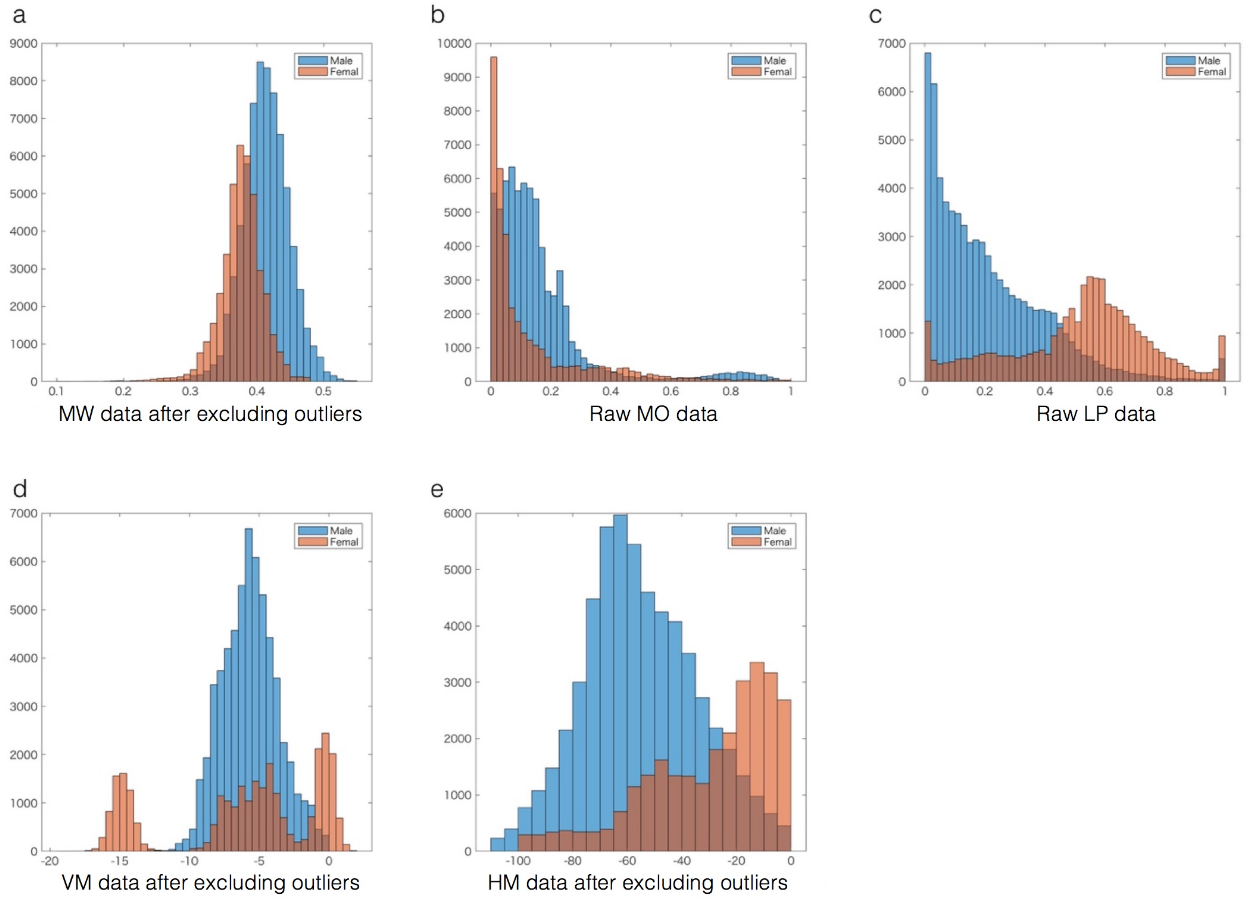

Supplementary Figure S2. Histograms for each parameter measured in male and female groups. Distributions of total recorded data are shown as histograms. These data included all data collected during measurements, which were not time-locked to swallowing. However, MW (a), VM (d), and HM (e) data were modified after the exclusion of outliers. MO (b) and LP (c) data are presented as raw data. Blue bins were obtained from all five male participants, and red bins were calculated from all five female participants. For each parameter, we used the Wilcoxon rank sum test to reveal differences between these two groups.

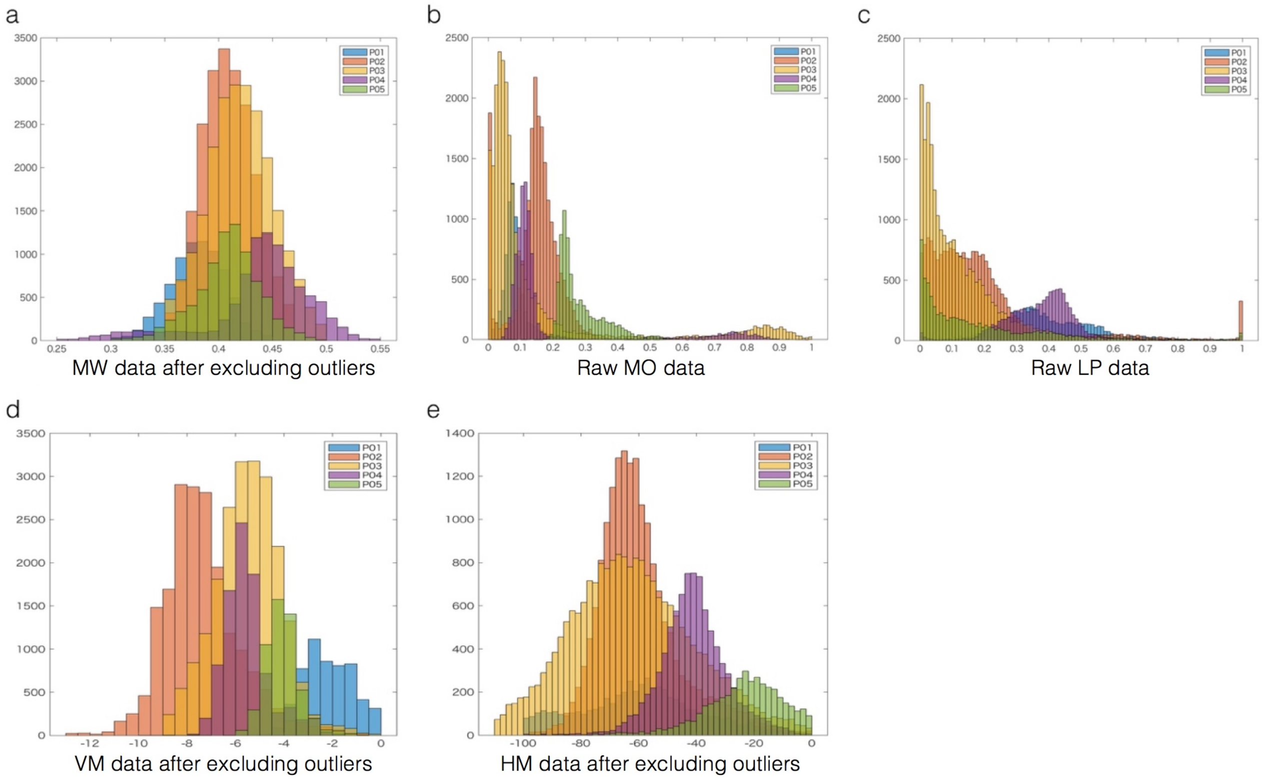

Supplementary Figure S3. Histograms for each parameter measured in individual males. Distributions of total recorded data between individual males are shown as histograms for each parameter. These data included all data collected during measurements, which were not time-locked to swallowing. However, MW (a), VM (d), and HM (e) data were modified after the exclusion of outliers. MO (b) and LP (c) data are presented as raw data. P01 is Participant 1; designations for other participants used the same numbering scheme. The numbers of each participant correspond to the numbers in Table 1. For each parameter, we used the Kruskal-Wallis test to determine differences among the five individuals.

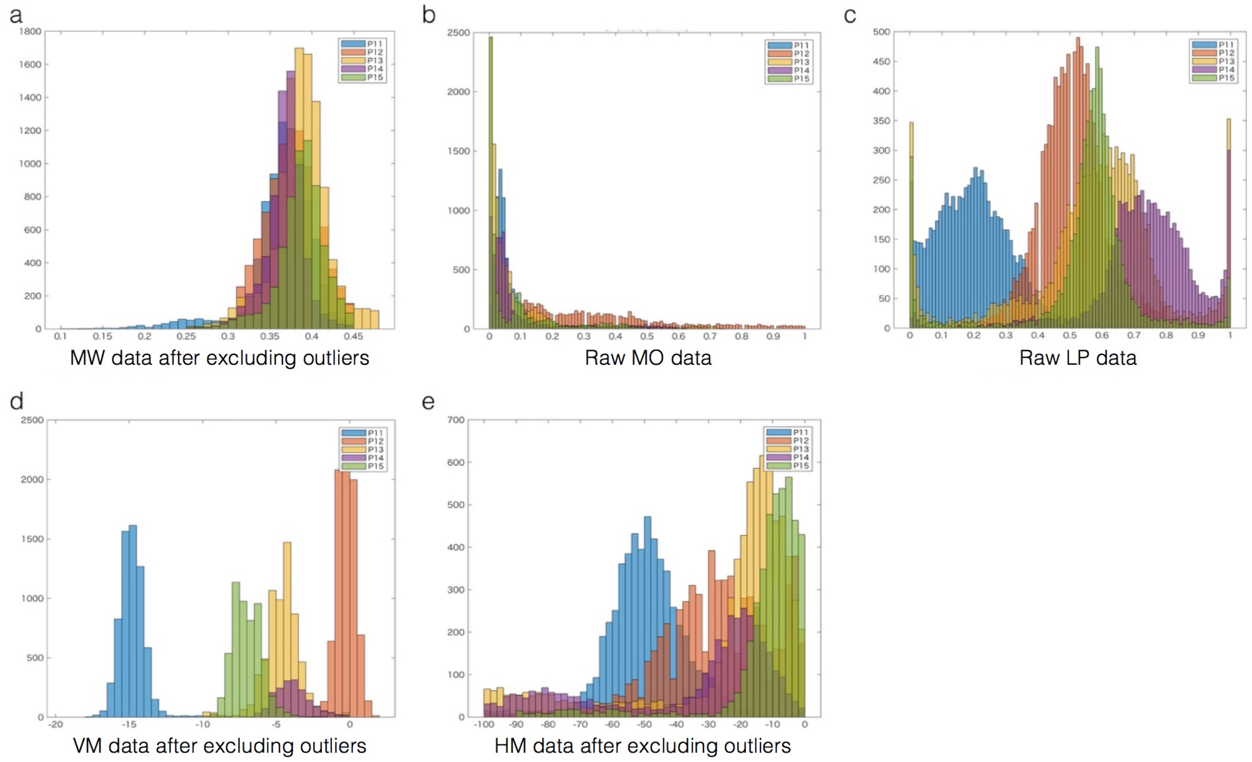

Supplementary Figure S4. Histograms for each parameter measured in individual females. Distributions of total recorded data from individual females are shown as histograms for each parameter. These data included all data collected during measurements, which were not time-locked to swallowing. However, MW (a), VM (d), and HM (e) data were modified after the exclusion of outliers. MO (b) and LP (c) data are presented as raw data. P11 is Participant 11; designations of other participants use the same numbering scheme, and participant numbers correspond to the numbers in Table 1. For each parameter, we used the Kruskal-Wallis test to reveal differences among the five individuals.

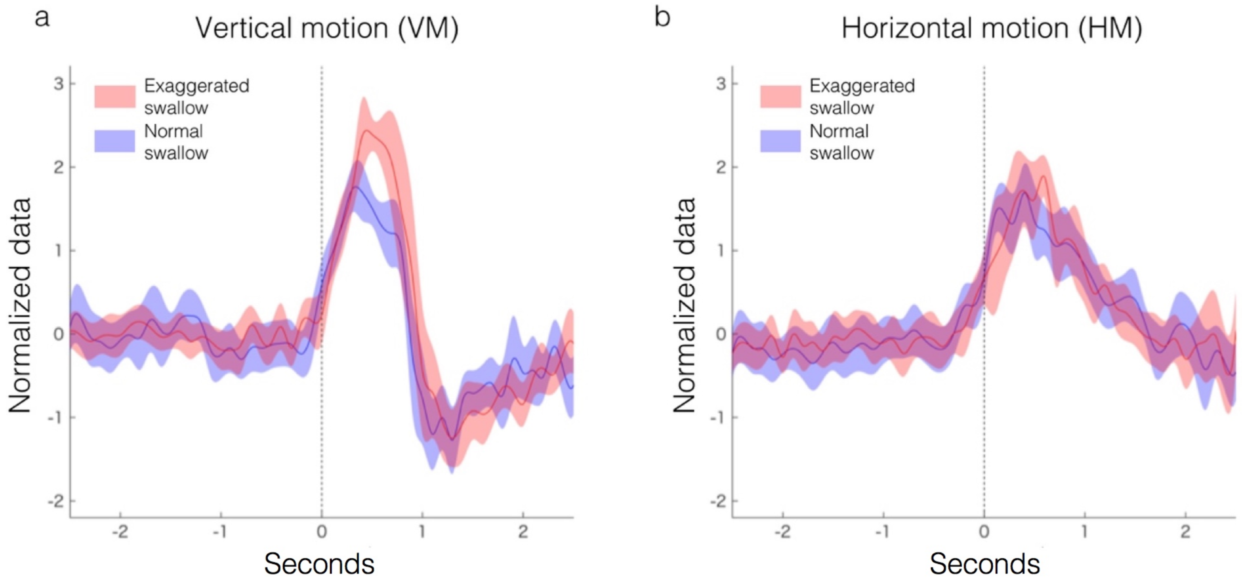

Supplementary Figure S5. Graphs of larynx-related parameters during normal swallowing and exaggerated swallowing in Participant 1. (a) Normalized temporal data for VM. (b) Normalized temporal data for HM. These plots were obtained from a representative subject, Participant 1. We assessed the influence of body motion on larynx-related parameters because auxiliary movements were commonly observed during swallowing. Normal swallow means that the participant only swallowed without other body motions. Exaggerated swallow means that the participant swallowed with a supporting swallow motion, which included straightening his back, bending himself backward and pulling in his chin. The range of motion was slight and within approximately 5 cm. The error bars indicate 95% CIs. Zero seconds corresponds to the onset of swallowing. An obvious difference in HM was not observed between the two conditions, but a slightly greater change in VM after swallowing occurred during the exaggerated swallow than during the normal swallow. We inferred that the VM was influenced by body motion, particularly bending backward. Bending backward is a rolling motion in the yz plane, but the VM was calculated using only coordinates from the xy plane. Therefore, the VM was influenced by bending backward because the motion occurring in the yz plane was not corrected.

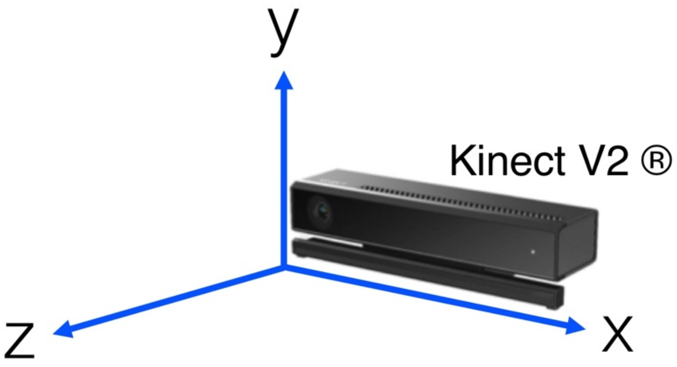

Supplementary Figure S6. The xyz axes provided by the Kinect v2. The Kinect v2 possesses infrared depth sensors, and it recognizes body parts in real time and simultaneously provides the x, y, and z spatial coordinates.

Participant	MW			VM			HM		
	upper limit	lower limit	exclusion rate	upper limit	lower limit	exclusion rate	upper limit	lower limit	exclusion rate
1	0.45	0.3	2.8%	0	-6	9.6%	0	-100	9.8%
2	0.47	0.35	4.5%	0	-13	2.5%	0	-100	5.3%
3	0.5	0.35	6.1%	0	-9	5.8%	0	-110	4.1%
4	0.55	0.25	0.2%	0	-10	0.9%	0	-100	1.0%
5	0.5	0.3	0.7%	0	-10	13.1%	0	-100	26.7%
11	0.45	0.1	0.6%	-10	-20	6.0%	0	-100	9.7%
12	0.45	0.3	9.5%	2	-4	2.1%	0	-100	6.1%
13	0.48	0.25	1.5%	0	-10	24.2%	0	-100	15.1%
14	0.45	0.25	2.0%	0	-10	50.6%	0	-100	16.4%
15	0.45	0.25	7.7%	0	-10	11.6%	0	-90	26.7%

Supplementary Table

Supplementary Table S1. Lists of upper and lower limits for MW, VM, and HM. Participant numbers correspond to the participant numbers in Table 1. We set upper and lower limits to exclude outliers from the raw MW, VM, and HM data. The exclusion rate indicates how many outliers were excluded from the total raw data.

Supplementary Video

Supplementary Video S1. Video of motions obtained from Participant 1 during the period ranging from 2.5 s before swallowing to 2.5 s after swallowing and simultaneously recorded waveforms. A, The video shows the motion of the mouth and the larynx of Participant 1 from 2.5 s before swallowing to 2.5 s after swallowing. The video was recorded by the Kinect RGB camera. **B,** Schematic of the video recording system is shown. Three blue stickers were attached to the thyroid cartilage to obtain the larynx-related parameters. **a,** Waveforms were recorded by a laryngograph (EGG). **b,** Waveforms were recorded by a throat microphone. **c,** The mouth-related parameters MW, MO, and LP are shown. **d,** The larynx-related parameters VM and HM are shown. All four waveforms show the average data and are the same as those shown in Figure 3.
